# Retinal OCT Findings in Patients after COVID Infection

**DOI:** 10.3390/jcm10153233

**Published:** 2021-07-22

**Authors:** Dominika Szkodny, Edward Wylęgała, Paulina Sujka-Franczak, Edyta Chlasta-Twardzik, Rafał Fiolka, Tomasz Tomczyk, Adam Wylęgała

**Affiliations:** 1 Chair and Clinical Department of Ophthalmology, Faculty of Medical Sciences, Zabrze Medical University of Silesia in Katowice, 40-760 Katowice, Poland; wylegala@gmail.com (E.W.); edyta.chlasta@gmail.com (E.C.-T.); fiolkarafal@gmail.com (R.F.); adam.wylegala@gmail.com (A.W.); 2 Department of Ophthalmology, District Railway Hospital in Katowice, 40-760 Katowice, Poland; paulina.s.franczak@gmail.com; 3 Temporary Hospital of Health Care Facility of the Ministry of the Interior and Administration in Katowice, 40-163 Katowice, Poland; t.tomczyk@icloud.com

**Keywords:** COVID-19, optical coherence tomography, angiography, retina, choroid, vascular complications

## Abstract

Purpose: The aim of this study was to assess and compare the optic nerve, retina, and retinal vessel parameters in recovered COVID-19 patients and healthy patients by using optical coherence tomography angiography (OCT-a). Methods: In all, 156 eyes of post-COVID-19 patients and 98 eyes of subjects from a control group were enrolled in our study. BCVA, intra ocular pressure (IOP) measurement, fundus examination, and OCT images, including macular cube, OCT-RNFL, and angio-OCT 6 × 6 mm examinations, were performed for both groups. The measurements were acquired using Swept Source OCT DRI OCT Triton. In the post-COVID-19 group, 762 OCT protocols were obtained. For statistical analysis, parameters from only one eye from each subject were taken. Results: In the measured parameters, no significant differences were observed, i.e., central macular thickness (*p* = 0.249); RNFL (*p* = 0.104); FAZ (*p* = 0.63); and vessel density of superficial retinal vascular plexus in central (*p* = 0.799), superior (*p* = 0.767), inferior (*p* = 0.526), nasal (*p* = 0.402), and temporal (*p* = 0.582) quadrants. Furthermore, a slit-lamp examination did not reveal any COVID-19-related abnormalities. Conclusion: OCT examination did not detect any significant changes in morphology or morphometry of the optic nerve, retina, or the retina vessels due to COVID-19.

## 1. Introduction

The novel coronavirus called SARS-CoV-2 was detected for the first time in December 2019 in Wuhan, Hubei province, in the People’s Republic of China. The first patient with confirmed SARS-CoV-2 infection in Poland was reported in March 2020. Increase in new cases of the disease in our country has been observed from October, and currently we have 1,828,313 cases confirmed. Although respiratory manifestations mainly characterize COVID-19, various extrapulmonary symptoms have been also described. These include among others gastrointestinal, cardiovascular, neurological, dermatological, renal, and ocular complications [1,2,3]. In the literature, it has been documented that ACE2 is a receptor for SARS-CoV-2. From an ophthalmological point of view, this is significant because of the presence of ACE and ACE2 receptors in the choroid and the retina [4]. Ocular conditions during the course of COVID-19 infection are relatively rare, with a low prevalence of symptoms related to the anterior segment of the eye, such as dry eyes, foreign-body sensation, conjunctivitis, redness, and blurry vision [5]. However, more severe conditions related to the retina and retina vessels have also been described, mainly as case reports.

Few studies have been performed to assess the microvascular findings in the choroid and retina of patients who have been diagnosed with COVID-19. Optical coherence tomography (OCT) is a noninvasive imaging technique that allows one to obtain detailed images of the retina. For several years, it has been possible to visualize retinal and choroidal vasculature, without the use of contrast, due to the development of OCT angiography [6,7]. Recently, scientific reports have been published describing abnormalities such as an increase in RNFLT, retinal vessel enlargement, and lower vessel density of the superficial and deep retinal capillary plexus in patients after SARS-CoV-2 infection compared to a control group [4,8,9,10]. This study aimed to assess the presence of possible ophthalmological complications after symptomatic coronavirus disease 2019 by appraising the morphology and morphometry of the optic nerve, retina, and retinal vessels using optical coherence tomography in a group of post-COVID-19 subjects.

## 2. Materials and Methods

**Figure 1:**
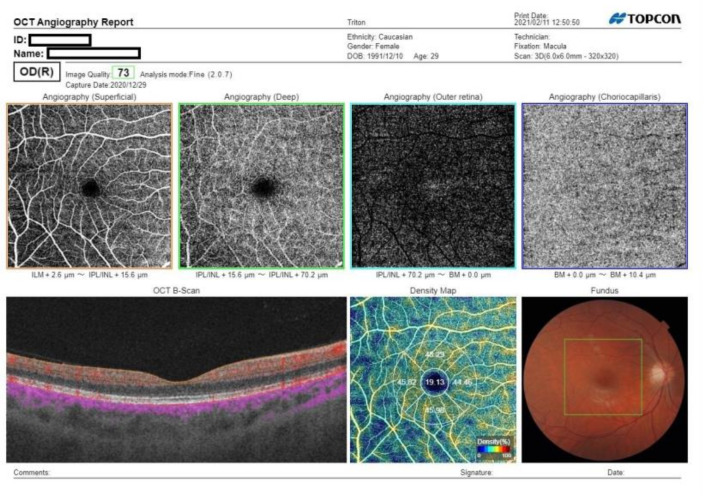
Optical coherence tomography angiograms of a patient, one month from COVID-19 onset. There are no remarkable abnormalities present.

In December 2020, following the Helsinki Declaration, this cross-sectional study was approved by the bioethical committee of Silesian Medical University. Adult patients with a history of symptomatic SARS-CoV-2 infection, confirmed by a positive test result via PCR of a nasopharyngeal swab sample and a recovery time of 1–4 months were enrolled, including 46 outpatients and 32 hospitalized patients. Patients aged under 18, with a history of symptomatic SARS-CoV-2 infection without a positive PCR test result; with severe general conditions, including acute respiratory distress syndrome (ARDS), myocarditis, cardiac arrhythmia, respiratory insufficiency, and kidney or multiple organ failure; or unable to take part in the study were excluded. Examinations were performed after isolation due to COVID-19 disease or after hospitalization in the COVID-19 department and conducted in the ophthalmology department of the same hospital. For each patient, a detailed medical history including ophthalmic data, systemic diseases, ocular symptoms, hospitalization, and oxygen supplementation during infection of SARS-CoV-2 was obtained. The PCR result was either provided by the patient before examination or was available in their medical history from the hospital. The control group consisted of healthy students and employees of the Railway Hospital in Katowice without a history of symptoms indicating COVID-19 infection and with a negative PCR test result, which was performed for all hospital staff in November 2020. Both groups underwent complete ophthalmic examination, including a visual acuity test, which was measured in a logMAR scale, intra ocular pressure (IOP) measurement, slit-lamp examination, OCT of the macula and optic nerve, and angio-OCT. For OCT scans, only patients with a transparent optical media and who had not been diagnosed with ophthalmological conditions were included. All scans were acquired with Swept Source OCT-DRI OCT Triton (Topcon Inc., Tokyo, Japan). OCT protocols included 3D macula −7 × 7 cube scan, 3D Disc −6 × 6 cube scan, and 6 × 6 macular angio-OCT (Figure 1). SCP vessel density was assessed using IMAGEnet 6 software. Each scan was assessed in order to detect any deviation in morphology or morphometry of the optic nerve, macula, and retinal vessels. The FAZ area was assessed manually by two graders. The arithmetic average was taken from both measurements. The density of the superficial vascular plexus, retinal nerve fiber layer, and central macular thickness were analyzed in comparison to those of the control group.

## 3. Statistical Analysis

Statistical analysis was completed using Statistica 13.3 (Tibco, Palo Alto, CA, USA). The mean standard deviation was computed for each variable after testing for normality of the distribution using the Shapiro–Wilk test. Differences between COVID-19 and control groups were tested using the Mann–Whitney U Test. The *t*-test for independent samples was applied to test between RNFL, CMT, and SPD between the groups. Linear regression analysis was performed between days from COVID-19, oxygen supplementation and hospitalization, and other parameters. Moreover, *p* < 0.05 was considered statistically significant. Parameters from only one eye from each subject were analyzed.

## 4. Results

This study comprised 254 eyes. A total of 156 eyes were included in the study group, while 98 eyes were included in the control group. In all, 27 men and 51 women who had been infected with COVID-19 participated in the analysis. While the control group consisted of 23 and 26 males and females, respectively. The mean age ±SD of participants was 49.9.3 ± 12.78. There was no difference in the age of men vs. that of women in our study (50.57 ± 1.48 vs. 48.9 ± 1.9 years, *p* = 0.640).

### Study Group Results

In the COVID-19 group, oxygen was supplemented to 26 patients, while 46 patients were not hospitalized (Table 1). All of patients requiring oxygen supplementation suffered from pneumonia and fever, 14 hospitalized patients and 11 unhospitalized ones reported anosmia and ageusia. We found a systemic association in the study group. One patient was diagnosed with myasthenia, two had type 2 diabetes, 14 patients had hypertension, and four had thyroid diseases (two Hashmito, one Graves–Basedow, and one hyperthyroid), one patient had restrictive obstructive lung disease. The mean IOP was 14.5 ± 0.19, while the mean BCVA (logMar) was 0.05 ± 0.15. Indirect ophthalmoscopy revealed fundus changes in nine patients: one participant had myopic fundus; one had vessel tortuosity; four had dry AMD with multiple drusen; two had peripapillary atrophy, one had glaucoma. The patient with vessel tortuosity did not report any clinical signs during COVID-19. The occurrence of any abnormalities in the optic nerve and retinal morphology, such as a cotton wool appearance, hemorrhages, macular and papilledema, vasculitis, epiretinal membrane retinal vein occlusion, or central retinal artery occlusion in any patient, was not confirmed by indirect fundus ophthalmoscopy. During COVID-19, one patient suffered from episcleritis, two had conjunctivitis, three complained of unspecified eye pain, and one had visual disturbances. None of the control patients reported any ocular surface problems during this period. In the OCT examination, 254 eyes were included, the remainder were excluded due to poor quality scans. For all included eyes, three different OCT protocols were performed, in total resulting in 762 OCT examinations. For the statistical analysis, 381 OCT scans were selected (Table 2).

The differences between groups for all tested parameters were insignificant, i.e., FAZ, *p* = 0.211; SPD central, *p* = 0.145; SPD superior, *p* = 0.507; SPD inferior, *p* = 0.828; SPD nasal, *p* = 0.23; SPD temporal, *p* = 0.324; CMT, *p* = 0.377; and RNFL, *p* = 0.52 (Table 2).

## 5. Correlations

Table 3 summarizes the results of the regression analysis for all measured parameters. CMT showed the most pronounced correlation with gender (R = −0.57, *p* = 0.01), and SPD superior (R = −0.51, *p* = 0.02). Oxygen supplementation was highly correlated with hospitalization (R = 0.73, *p* < 0.001) and BCVA logMAR (R = 0.45, *p* = 0.46). RNFL thickness corelated inversely with IOP (R = −0.48, *p* = 0.033). CMT showed a medium correlation with SPD superior (R = −0.51, *p* = 0.022). SPD parameters correlated with each other; the inferior with the superior (*p* = 0.001, R = 0.7), the superior with the temporal (*p* = 0.027, R = 0.49), and the inferior with the temporal (R = 0.5, *p* = 0.026) (Table 3).

## 6. Discussion

Since the outbreak of the COVID-19 pandemic, reports of various complications beyond those related to the respiratory system have been published. The presence of the SARS-CoV-2 virus in tears of infected patients has been demonstrated, and the pathogen is known to cause ocular-surface disorders such as chemosis, epiphora conjunctival congestion, mild eyelid edema, a burning eye sensation, foreign body sensation, and episcleritis with a very varied frequency of ocular findings having been detected in different studies [5,11,12,13,14]. Some of these symptoms were also observed in our study and are similar to common ocular conditions with viral etiologies.

Furthermore, there is some dispute as to whether SARS-CoV-2 affects the retina and choroid, taking into consideration the fact that ACE2, which is a receptor for the virus, has been identified in these structures [15,16,17]. After the outbreak of the pandemic, retinal and vascular pathologies in patients with coronavirus disease 2019 were rapidly reported [18,19]. Marinho et al. described hyperreflective lesions at the level of the ganglion cells and inner plexiform layer, observed via OCT. Additionally, in this study, four patients presented subtle cotton wool spots and microhemorrhages along the retinal arcade [19]. The interpretation of these abnormalities is limited by the undefined mean and median age of patients and the lack of data on comorbidities. Furthermore, the authors did not report any abnormalities in the OCT data, and it is possible that the retinal alterations may be a result of nerve myelination [20]. Acute vascular lesions of the inner retina, including flame-shaped hemorrhages and cotton wool spots, have been also observed in fundus examination of patients during hospitalization due to severe COVID-19 [18]. Due to the presence of concomitant diseases such as diabetes and hypertension, it is unclear whether these findings might be caused directly by SARS-CoV-2 infection. Only some cases had no comorbidities, and authors suggest that in these patients, retinal lesions might be related to COVID-19 itself. Contrary to these studies, we did not notice any similar retinal abnormalities in OCT and fundus examinations of patients with systemic diseases or in the rest of the patients. In the study group, clinical signs collected from the OCT included eye disorders not connected with coronaviruses such as AMD, peripapillary atrophy, and myopia. Vessel tortuosity was most likely unrelated to COVID-19, since the patient have not reported any deterioration of visual acuity or systemic symptoms during the disease.

In previously published studies, connections between rare ocular conditions such as inflammatory chorioretinopathy, serpiginous choroiditis, central retinal artery occlusion, optic neuritis, and impending central retinal vein occlusion have also been described [15,16,21,22,23]. All of these disorders have been reported as single cases and have not been observed on a larger scale. Whether or not these findings represent coincidental relationships remains equivocal.

Due to the increased risk of blood clotting disorders present during COVID-19 infection, there are speculations related to whether SARS-CoV-2 causes asymptomatic ocular microangiopathic syndrome [24]. In a recent study, Abrishami et al. identified the mean macular SCP, VD, and DCP vessel density reduction and FAZ area increase in the COVID-19 cohort versus age-matched controls [4]. Additionally, Savastano et al. noticed a reduced perfusion density of the radial peripapillary capillary plexus in recovered COVID-19 patients compared to that in a control group [25]. Moreover, in a study performed on 90 recovered COVID-19 patients, the authors described an increase in peripapillary RNFLT and macular GCC compared to controls, suggesting that the virus may also affect the optic nerve [10]. Despite these reports of the retinal capillary microvasculature changes due to SARS-CoV-2 infection, in our study, we did not confirm any differences between the study and control groups in terms of superficial vessel plexus density, FAZ area, central macular thickness, or RNFL thickness. Furthermore, there was no correlation between angio-OCT parameters and oxygen supplementation or hospitalization. Correlations presented in our study, including inverse correlation between RNFL with IOP and positive correlation between retinal blood supply and retinal thickness, have also been demonstrated in other studies [26,27].

There are several limitations to our study. The major limitation is the different severity of the disease, which may have influenced the results. Furthermore, we did not possess the results of examinations taken prior to the onset of disease. Therefore, we were not able to ascertain whether OCT measurements changed after COVID-19 infection. Additionally, we were not able to examine patients during infection; therefore, some ocular symptoms could have been missed. However, taking into account the fact that in 1–4 months we have not found any changes in OCT, it is also reasonable to assume that these patients did not have abnormalities before the disease, and if there were any changes during infection, it was probably mild and did not leave any permanent signs.

To conclude, in our study, we did not observe any ocular disturbances caused by COVID-19. In light of the different studies already published that have presented OCT and fundoscopic examination data, it is difficult to explicitly state whether SARS-CoV-2 is responsible for any changes in the choroidal or retinal vasculature. Therefore, further investigations with more patients are needed, especially focused on the active phase of the disease.

## Figures and Tables

**Table 1:** Qualitative demographic data.

	Number of Eyes	
**COVID**	**78**	
Control	49	
	**Oxygen supplementation**	
Yes	26	
No	52	
	**Hospitalization**	
Yes	26	
No	52	
	**Gender**	
	Male	Female
COVID	27	51
Control	23	26

**Table 2:** Descriptive statistics for groups and *t*-test *p*-value.

	Mean	Std. Err	−95	95	*p* Value
RNFL					
COVID	107.24	1.6217	104.02	110.46	
Control	100.49	2.1056	96.30	104.67	0.104
CMT					
COVID	253.69	3.8282	246.09	261.30	
Control	267.51	4.9704	257.64	277.39	0.249
Angio-oct-SPD central					
COVID	20.291	0.3910	19.514	21.067	
Control	19.755	0.5076	18.747	20.763	0.799
SPD-superior					
COVID	48.497	0.3407	47.821	49.174	
Control	49.438	0.4423	48.560	50.316	0.767
SPD-inferior					
COVID	47.767	0.4684	46.836	48.697	
Control	48.309	0.6081	47.101	49.517	0.526
SPD-nasal					
COVID	45.800	0.3073	45.189	46.410	
Control	45.679	0.3990	44.886	46.471	0.403
SPD-temporal					
COVID	46.64	0.48	45.69	47.59	
Control	46.54	0.47	45.60	47.48	0.582
FAZ					
COVID	354.64	15.038	324.78	384.51	
Control	322.71	19.525	283.93	361.49	0.063
Age					
COVID	50.577	1.4776	47.630	53.524	
Control	48.898	1.9009	45.076	52.720	0.640
Weeks from COVID					
COVID Control	6.360000	0.540817	5.282399	7.437601	
BCVA					
COVID	0.92	0.02	0.88	0.96	
Control	0.99	0.02	0.95	1.04	0.220
BCVA logMAR					
COVID	0.07	0.02	0.02	0.11	
Control	0.00	0.02	−0.04	0.04	0.290
IOP					
COVID	14.61	0.32	13.97	15.26	
Control	14.91	0.32	14.27	15.55	0.330

**Table 3:** Pearson correlation coefficient matrix for IOP; fundus alterations; RNFL; CMT; OCT-a—central, superior, nasal, inferior, and temporal; FAZ; gender; weeks post COVID-19; oxygen supplementation; and hospitalization. Statistically significant correlations are in bold.

Variable	Age	sd Inferior	Gender	Weeks from COVID	Oxygen	Hos.	BCVA logMAR	IOP	Alterations	RNFL	CMT	Angio-oct-SPD Central	SPD-Superior	SPD-Inferior	SPD-Nasal	SPD-Temporal	FAZ
Age	-	0.41	0.26	0.38	0.20	0.15	0.33	−0.23	0.14	0.13	−0.10	−0.20	−0.09	0.06	−0.18	0.01	0.14
sd inferior	0.41	-	0.33	0.09	0.11	−0.04	0.19	−0.23	0.34	−0.23	−0.04	0.11	−0.20	−0.07	0.05	**−0.50**	**−0.62**
Gender	0.26	0.33	-	0.06	0.24	0.18	0.12	−0.08	0.22	−0.16	**−0.57**	−0.20	0.29	0.14	0.39	0.21	−0.20
Weeks from covid	0.38	0.09	0.06	-	−0.24	0.00	−0.27	−0.32	0.12	−0.06	−0.23	0.05	−0.12	−0.17	0.23	0.06	−0.17
Oxygen	0.20	0.11	0.24	−0.24	-	**0.73**	**0.51**	−0.03	−0.03	0.07	−0.22	−0.12	0.09	−0.22	−0.27	−0.19	−0.03
Hos.	0.15	−0.04	0.18	0.00	**0.73**	-	0.08	0.08	0.11	0.00	−0.10	−0.31	−0.19	−0.27	−0.41	−0.14	0.13
BCVA logMAR	0.33	0.19	0.12	−0.27	**0.51**	0.08	-	0.03	−0.15	0.19	0.00	0.32	0.11	−0.04	−0.06	0.06	−0.14
IOP	−0.23	−0.23	−0.08	−0.32	−0.03	0.08	0.03	-	0.19	**−0.48**	0.13	0.06	−0.01	0.08	−0.04	0.32	0.44
alterations	0.14	0.34	0.22	0.12	−0.03	0.11	−0.15	0.19	-	−0.28	−0.27	−0.08	−0.19	0.08	−0.07	−0.07	−0.18
RNFL	0.13	−0.23	−0.16	−0.06	0.07	0.00	0.19	**−0.48**	−0.28	-	0.36	0.07	−0.15	−0.01	−0.09	0.18	0.12
CMT	−0.10	−0.04	**−0.57**	−0.23	−0.22	−0.10	0.00	0.13	−0.27	0.36	-	0.06	**−0.51**	−0.13	−0.14	−0.04	0.25
angio-oct-SPD central	−0.20	0.11	−0.20	0.05	−0.12	−0.31	0.32	0.06	−0.08	0.07	0.06	-	0.10	0.10	0.29	0.05	−0.39
SPD-superior	−0.09	−0.20	0.29	−0.12	0.09	−0.19	0.11	−0.01	−0.19	−0.15	**−0.51**	0.10	-	**0.69**	0.38	**0.49**	−0.10
SPD-inferior	0.06	−0.07	0.14	−0.17	−0.22	−0.27	−0.04	0.08	0.08	−0.01	−0.13	0.10	**0.69**	-	0.31	**0.50**	0.03
SPD-nasal	−0.18	0.05	0.39	0.23	−0.27	−0.41	−0.06	−0.04	−0.07	−0.09	−0.14	0.29	0.38	0.31	-	0.42	−0.34
SPD-temporal	0.01	**−0.50**	0.21	0.06	−0.19	−0.14	0.06	0.32	−0.07	0.18	−0.04	0.05	**0.49**	**0.50**	0.42	-	0.39
FAZ	0.14	**−0.62**	−0.20	−0.17	−0.03	0.13	−0.14	0.44	−0.18	0.12	0.25	−0.39	−0.10	0.03	−0.34	0.39	-

IOP—intraocular pressure, FAZ—foveal avascular zone, Hos.—hospitalization.

## Data Availability

Data are available on request due to restrictions, e.g., privacy or ethical concerns.

## Abbreviations

BCVA—best corrected visual acuity; IOP—intraocular pressure; SPD—superficial plexus density; FAZ—foveal avascular zone; CMT—central macular thickness; OCT-a—OCT angiography; RNFL—retinal nerve fiber layer.
